# Philadelphia Hospital

**Published:** 1839-04-06

**Authors:** 


### CLINICAL LECTURE.

### PHILADELPHIA HOSPITAL.

### ON PNEUMONIA—(CONTINUED.)

*Saturday, January 19th.*—Dr. GERHARD commenced as follows:

In my last lecture I pointed out to you a number of cases of simple pneumonia, terminating, as such cases generally do, in complete recovery. When acute pneumonia takes an unfavourable turn, it is usually from the complication of inflammation of other organs than the lungs. In certain rare cases, it is true, that the disease may terminate fatally by the mere extent of lung which is involved; and then the patient perishes from the obstruction to the respiration, a large portion of the pulmonary tissue gradually becomes impermeable to the air, and prevents the necessary change in the blood. But such cases are rare. As an ordinary rule, we may, therefore, regard the cause of death, in inflammatory pneumonia, to depend upon the concurrent inflammation of other organs essential to life. If a single one of these organs be inflamed, the probability of a fatal result is comparatively slight; but the probability of death is vastly increased, when the inflammation of these organs is superadded to that of the lungs.

Pneumonia offers, at certain seasons, an unusual tendency to these complications; in fact, we meet with very few simple cases of the disease; this tendency sometimes manifests itself in several different ways. In all cases, there is undoubtedly some unknown cause which gives rise to an inflammatory diathesis; but the local lesions themselves may either occur simultaneously with the most important inflammation, which is seated in the lungs, or they may be developed at a much later period, when the disease of the lungs has already passed through its early stage. In this case, the lesions of other organs are said to be strictly secondary. When the inflammatory complications of pneumonia occur at a very early period, the disease should be looked upon as a general, instead of a local affection; and we may regard its cause to be the altered state of the blood, which here assumes, in the most strongly-marked manner, the characteristic features of the inflammatory diathesis. Perhaps the modification of the blood will be found to depend upon some previous change of the nervous power, and this may constitute the second, instead of the first link, in the chain of morbid actions; but it will be, at present, convenient for you to regard it as the earliest phenomenon, or, at least, as one of the earliest phenomena occurring in many cases of pneumonia. The alteration of the blood is nearly proportioned to the number and severity of the lesions which complicate pneumonia; and the thick, buffy coat, and firm, cupped coagulum, are, as a general rule, much more evident when the inflammation extends to several organs. Certain organs, however, give rise to more evident inflammatory appearances than others; this is particularly the case with the heart and larger arteries, which are often inflamed in severe cases of pneumonia, and cause the most widely spread secondary inflammations.

The tendency of the organs of the body to take on inflammatory action in pneumonia, it will probably be found, is regulated by fixed rules; we have not, however, at present, all the facts necessary for us to establish the exact degree of frequency with which the organs are inflamed; we may, however, approach an accurate result. The experience of this winter has nearly coincided with my usual observations; but, in this respect, you must not expect a considerable degree of variety. In one year a certain organ may be much more frequently diseased than in another, and epidemics of pneumonia often appear which are characterized by these organic inflammations. Such is the case with the well-known bilious pneumonia described by Stoll, at Vienna, and with several epidemics in which the brain was principally affected.

The organs most disposed to take on inflammation in pneumonia, are the heart and its membranes, the brain, and the liver. When you examine a very large number of cases without reference to particular seasons, you will find that the liver is, on the whole, most frequently inflamed. This inflammation, it would seem, depends chiefly upon mere contiguity, and nearly always takes place when the right lung, particularly its lower lobe, is inflamed. The disease is transmitted through the diaphragm, and the ordinary symptoms of jaundice are then superadded to those of pneumonia.

Next in frequency to the inflammation of the liver, and in certain seasons of much more frequent occurrence, is that of the heart. During the present course, you have seen numerous examples of this complication. The parts involved are the internal membrane, including the valves, the pericardium, and, perhaps, (although of this fact the proof must be somewhat doubtful,) the muscular substance of the heart. The part most frequently, or, at least, most seriously affected, is the lining membrane. This lesion is the more important, because it is attended with the stagnation of the blood in the heart, and the formation of those coagula, which increase the oppression of the patient, and accelerate the approach of death. I have observed many more cases of this lesion within the last year or two than at any previous period; the greater frequency was in part only apparent from the greater facility which I have acquired in recognising these lesions; but I have reason to believe that the disease is really more frequent than at any other period of my observations.

The next frequent complication of pneumonia, is inflammation of the brain, and of its membranes. This is a singular variety, and is the more interesting, because the symptoms of the pulmonary disease become obscure, and are sometimes lost during the increase of the cerebral symptoms. The signs of the cerebral are, in general, evident, but less intense than they appear to be in those cases in which the brain is primarily affected. I shall next point out to you such examples of these complications as may present themselves.

(To be continued.)

